# Phytochemical profiles and inhibitory effects of Tiger Milk mushroom (*Lignosus rhinocerus*) extract on ovalbumin-induced airway inflammation in a rodent model of asthma

**DOI:** 10.1186/s12906-016-1141-x

**Published:** 2016-06-03

**Authors:** M. Johnathan, S. H. Gan, M. F. Wan Ezumi, A. H. Faezahtul, A. A. Nurul

**Affiliations:** School of Dental Sciences, Universiti Sains Malaysia, 16150 Kubang Kerian, Kelantan Malaysia; Human Genome Centre, School of Medical Sciences, Universiti Sains Malaysia, 16150 Kubang Kerian, Kelantan Malaysia; School of Health Sciences, Universiti Sains Malaysia, 16150 Kubang Kerian, Kelantan Malaysia; Department of Pathology, School of Medical Sciences, Universiti Sains Malaysia, 16150 Kubang Kerian, Kelantan Malaysia

**Keywords:** *Lignosus rhinocerus*, Airway inflammation, Asthma, GC-MS, Linoleic acid, Tiger milk mushroom

## Abstract

**Background:**

*Lignosus rhinocerus* (*L. rhinocerus*), which is known locally as Tiger Milk mushroom, is traditionally used in the treatment of asthma by indigenous communities in Malaysia. However, to date, its efficacy on asthma has not been confirmed by scientific studies and there is also sparse information available on its active constituents. In this study, the volatile constituent of *L. rhinocerus* hot water extract was investigated using gas chromatography mass spectrometry (GC-MS). The potential effects of *L. rhinocerus* extract for anti-asthmatic activity was further investigated on ovalbumin (OVA)-sensitized asthmatic Sprague Dawley rats.

**Methods:**

Sequential extraction using five solvents (petroleum ether, diethyl ether, hexane, ethyl acetate and methanol) was conducted prior to GC-MS analysis. Male Sprague Dawley rats were divided into the following four groups of five animals each: 1) normal rats, 2) sensitization plus OVA-challenged rats 3) sensitization plus OVA-challenged with *L. rhinocerus* treatment and 4) sensitization plus OVA-challenged with dexamethasone treatment. The levels of immunoglobulin E (IgE) in the serum and T-helper 2 cytokines, including interleukin (IL)-4, IL-5 and IL-13, in bronchoalveolar lavage fluid (BALF), as well as eosinophil infiltration in the lungs, were investigated.

**Results:**

GC-MS analysis revealed the presence of five main groups (alkane, fatty acids, benzene, phenol and dicarboxylic acid) with a total of 18 constituents. Linoleic acid (21.35 %), octadecane (11.82 %) and 2,3-dihydroxypropyl elaidate (10.47 %) were present in high amounts. The extract significantly ameliorated the increase in total IgE in serum and IL-4, IL-5 and IL-13 levels in BALF and also effectively suppressed eosinophils numbers in BALF while attenuating eosinophil infiltrations in the lungs.

**Conclusion:**

*L. rhinocerus* hot water extract has the potential to be used as an alternative for the treatment of acute asthma.

## Background

Asthma affects 300 million people worldwide and is a result of a complex interplay between genetic and environmental factors [1]. Allergic asthma is a chronic disease characterized by recurrent wheezing, shortness of breath, chest tightness and coughing. The airway disorder is commonly characterized by airway eosinophilia infiltration, increased serum immunoglobulin E (IgE) and T-helper 2 (Th2) cytokine levels such as interleukin (IL)-4, IL-5 and IL-13 [2], as well as excessive production of airway mucus.

The Th2-dominated response is initially triggered when an airway allergen is taken by the dendritic cells and presented to specific Th cells by means of major histocompatibility complex (MHC) class II, thus stimulating the immunological synapse. Activated allergen-specific Th cells then polarize into Th1 or Th2 effector populations, differentiate and expand into a Th2 subpopulation. The activated Th2 cells function in recruiting and activating cytokines, including IL-4, IL-5 and IL-13, following stimulation of eosinophils. IL-4 cytokines have been reported to cause class switching of B cells, resulting in IgE synthesis, which is involved in mast cell degranulation [3]. The key role of IL-5 cytokines includes their involvement in the growth and differentiation of bone marrow eosinophils, as well as their release into the peripheral circulation [4]. Additionally, IL-5 also increases the activity of eosinophil recruitment, activation and survival at the sites of inflammation [5]. IL-13, which is known as the “central mediator of allergic asthma,” plays an important role in directing the allergic response in asthmatic patients [6]. Various studies are therefore primarily focused on products that have the potential to reduce IgE, IL-4, IL-5, IL-13 and eosinophils as therapeutic targets for asthma treatment [7–10].

Current asthma medications are mainly based on steroids and other types of anti-inflammatory drugs [11]. Among the available drugs, inhaled corticosteroids (ICSs) are very effective, as they suppress chronic airway inflammation in patients with asthma [12]. ICSs are generally considered harmless, though persistent use of ICSs at higher doses often leads to various systemic and local side effects [13], some of which include candidiasis, osteoporosis, growth retardation, cataracts and pharyngitis [14]. Thus, a safer alternative for the management of asthma is needed.

*Lignosus rhinocerus* (*L. rhinocerus*) from the *Polyporaceae* family is a wild mushroom exclusive to Malaysia. Locally known as “cendawan susu rimau” or “Tiger milk mushroom”, this unique mushroom is one of the reported 38 available types of edible mushrooms in Malaysia used for medicinal purposes by rural and indigenous communities [15]. The tuber is purported to contribute the most medicinal value, and the indigenous populations in Peninsular Malaysia utilise it to treat diseases such as asthma, fever, breast cancer, stomach cancer and food poisoning, as well as to heal wounds [15, 16]. Previous studies have demonstrated anti-proliferative activities [17] and immunomodulatory properties [18] of this mushroom sclerotial. A recent study by Lee et al. [19] reported anti-acute inflammatory properties of sclerotial powder of *L. rhinocerus* using carrageenan-induced paw oedema model in rats. In addition, the study also demonstrated a potent inhibition of TNF-α production by the high-molecular-weight fractions of the sclerotial powder of *L. rhinocerus*. However, to date, no studies have been reported on the anti-asthmatic properties of *L. rhinocerus* in airway inflammation models. Thus this study reported anti-asthmatic effects of *L. rhinocerus* sclerotial extract in ovalbumin-induced airway inflammation of the rodent model and the profile of volatile constituents of the mushroom extract by GCMS analysis.

## Methods

### Preparation of *L. rhinocerus* hot water extract

Sclerotia of *L. rhinocerus* cultivar TM02 was obtained in dried powdered form from Ligno Biotech Sdn. Bhd. (Selangor, Malaysia). The powder was subjected to hot water extraction using a soxhlet for 24 h and was further concentrated using a rotary evaporator (Unimax1010, Heidolph, Germany) before being freeze-dried in a freeze drier (Ilshin BioBase, Gyeonggi-do, Korea).

### Liquid-liquid extraction

A sequential liquid-liquid extraction was performed using 1) petroleum ether, 2) diethyl ether 3) hexane, 4) ethyl acetate and 5) methanol. The selected solvents ranged in polarity starting from non-polar (petroleum ether, diethyl ether and hexane) to more polar solvents (ethyl acetate and methanol) to take advantage of their different properties.

Briefly, 1 ml of petroleum ether was added to the capped glass tube containing 1 g of *L. rhinocerus* extract. The mixture was then vortexed for 1 min using a vortex mixer (Westbury, New York, USA), followed by centrifugation (Centrifuge Universal 32R, HettichZentrifugen, Germany) at 700 × g for 5 min. The supernatant was aspirated before being transferred (100 μl) to a new auto-sampler vial for GC-MS injection. The residue was used for subsequent extraction using diethyl ether followed by hexane, ethyl acetate and finally, methanol, as previously described for petroleum ether. Following extraction by each solvent type, the samples were individually injected into the GC-MS system in duplicate. Each sample was analyzed against a blank organic solvent containing a similar type of organic solvent used in the extraction process each time.

### GC-MS analysis

GC-MS analysis was performed on an HP6890 GC coupled with a HP5973 mass spectrometer (Hewlett Packard, CA, USA). The column was a HP-5MS fused-silica capillary column (50 m x 0.25 mm i.d.; 0.25-μm film thickness) with helium as the carrier gas, and it was run at a constant pressure of 9.78 psi. Injection was conducted using a splitless mode at an injector temperature of 250 °C. The oven temperature was ramped from 40 °C to 280 °C (1-min hold) at a rate of 25 °C/min. The oven temperature was held at 310 °C for 6 min for each analysis. The total run time for each sample was approximately 28 min. The GC-MS interface temperature was set to 280 °C. MS mode was used during analytical scanning from 20 to 650 atomic mass units (amu). The ion source temperature was set at 250 °C.

The blank was injected first each time, followed by the sample injection. The chromatograms obtained from the total ion current (TIC) were integrated without any correction for co-eluting peaks, and the results were expressed as total abundance. The TIC peaks and chromatograms were analyzed using an Agilent Technologies 7890A GC system with 597C VL MSD. All peaks were identified based on mass spectral matching (≥ 90 %) from both the National Institute of Standards and Technology (NIST) and Wiley libraries. Only compounds with 90 % or greater spectral matching accuracy were reported.

### Animals

Ethical approval was obtained from Animal Ethics Committee Universiti Sains Malaysia. Male Sprague Dawley rats (age between 6 and 8 weeks; 200–250 g in weight) were used for this experiment. The rats (*n* = 20) were housed in polystyrene cages in an air-controlled room. All animals were maintained at the animal house, and tap water was allowed ad libitum. The animals were acclimatized to the experimental environment prior to commencement of the study.

### Sensitization, airway challenge and treatment

The rats were divided into the following four groups of five animals each: (1) normal rats, (2) sensitization plus 1 % OVA challenge or “untreated group,” (3) sensitization plus 1 % OVA challenge with oral *L. rhinocerus* treatment (500 mg/kg per body weight) and (4) sensitization plus 1 % OVA challenge with intraperitoneal (ip) dexamethasone treatment (3 mg/kg per body weight). Sensitization on days 1 and 14 was conducted with a 2 × 1 ml i.p. injection of 10 mg/ml OVA (Nacalai Tesque, Kyoto, Japan) + 100 mg/ml alum (aluminium hydroxide) (Sigma, MO, USA) in phosphate buffered saline (PBS) (based on the modified method of Makhlouf and Hafez, [20]). An additional 50 ng/ml of *Bordetella pertussis* (Sigma, MO, USA) was also prepared with the solution as adjuvant. On day 23, the rats were challenged with 1 % OVA aerosol for 20 min/day for 7 days using an ultrasonic nebulizer (Mabist mist, Illinois, USA) and orally administered by gavage with 500 mg/kg per body weight of *L. rhinocerus* extract for 7 days. On day 30, 24 h after the final challenge, the rats were anesthetized with ketamine (100 mg/kg) and xylazine (10 mg/kg), and blood was collected by cardiac puncture.

### Eosinophils and inflammatory cell count

Following sacrifice, bronchoalveolar lavage fluid (BALF) was obtained using an endotracheal tube by instilling and aspirating the trachea with PBS solution. BALF was centrifuged at 500 × g for 5 min, and the pellet was re-suspended with PBS. The total inflammatory cell number was quantified by counting the number of cells from at least five squares of the haemocytometer after excluding dead cells stained with trypan blue.

### Measurement of total IgE in BALF

The total IgE was measured with a specific rat ELISA kit (Abnova, Heidelberg, Germany) according to the manufacturer’s instructions. IgE levels in each sample were measured from optical density readings at 450 nm followed by calculation based on a standard curve that was generated using recombinant IgE.

### Measurement of Th2 cytokines in BALF

An enzyme-linked immunosorbent assays (ELISA) was performed according to the manufacturer’s instructions. IL-4, IL-5 and IL-13 in BALF were measured using specific rat IL-4, IL-5 and IL-13 ELISA kits (CusaBio, Hubei, China). The bound enzyme that was proportional to the respective cytokines concentration was investigated at absorbance of 450 nm. Cytokine concentrations were calculated from standard curves that were generated using respective recombinant interleukins.

### Lung tissue histology

The lungs were removed, rinsed with PBS, weighed and fixed in 10 % formalin overnight for immunohistochemistry analysis. The lung tissue was embedded in paraffin and cut into 4-μm thickness sections, followed by haematoxylin and eosin staining for lung inflammation scoring. The tissue was subsequently mounted and cover-slipped with di-n-butyl phthalate in xylene (DPX) mounting medium. The infiltration intensity at peribronchial and perivascular inflammatory spaces was scored as the approximate number of cell layers around vessels or bronchioles following criteria adapted from Tournoy et al. [21]. Whole lung sections were assigned using a reproducible scoring system developed for a murine model (Curtis, et al., [22]). The scoring system ranges between 0 to 4 as follows, indicating inflammation severity based on no inflammation, the presence of occasional cuffs, the presence of a thin layer or presence of inflammatory cells surrounding the peribronchial/perivascular space that are 1–5 cells thick or > 5 cells thick:

- 0: no inflammation
- 1: mild inflammation
- 2: moderate inflammation
- 3: severe inflammation
- 4: extreme inflammation

For each rat, randomly distributed airway sections in the lung were analysed, and their mean scores were calculated. The inflammation present was evaluated with a double-blinded scoring method. Quantitative analysis of the lung inflammation score was performed using a Mirax Image viewer (Carl Zeiss, Jenna, Germany).

### Statistical analysis

Data are expressed as the mean ± standard deviation (SD). Statistical significance (*p* < 0.05) was determined by a one-way ANOVA followed by Scheffe’s post hoc correction and inflammation scores were analysed with Wilcoxon-Mann Whitney test using Statistical Program for Social Science (SPSS) version 22.0 (New York, USA).

## Results

### GC-MS analysis of the volatile constituent of *L. rhinocerus*

**Fig. 1:**
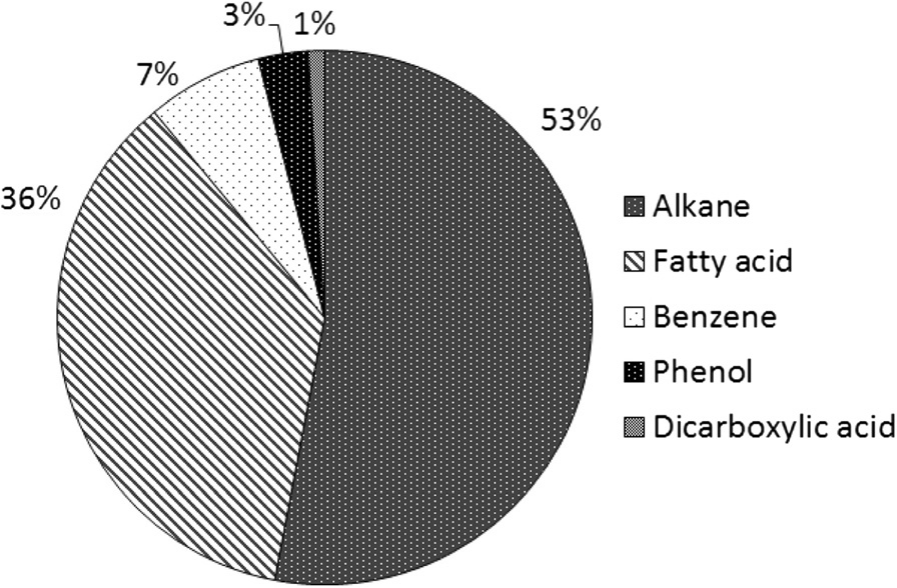
Groups of compounds present in *L. rhinocerus*

**Fig. 2:**
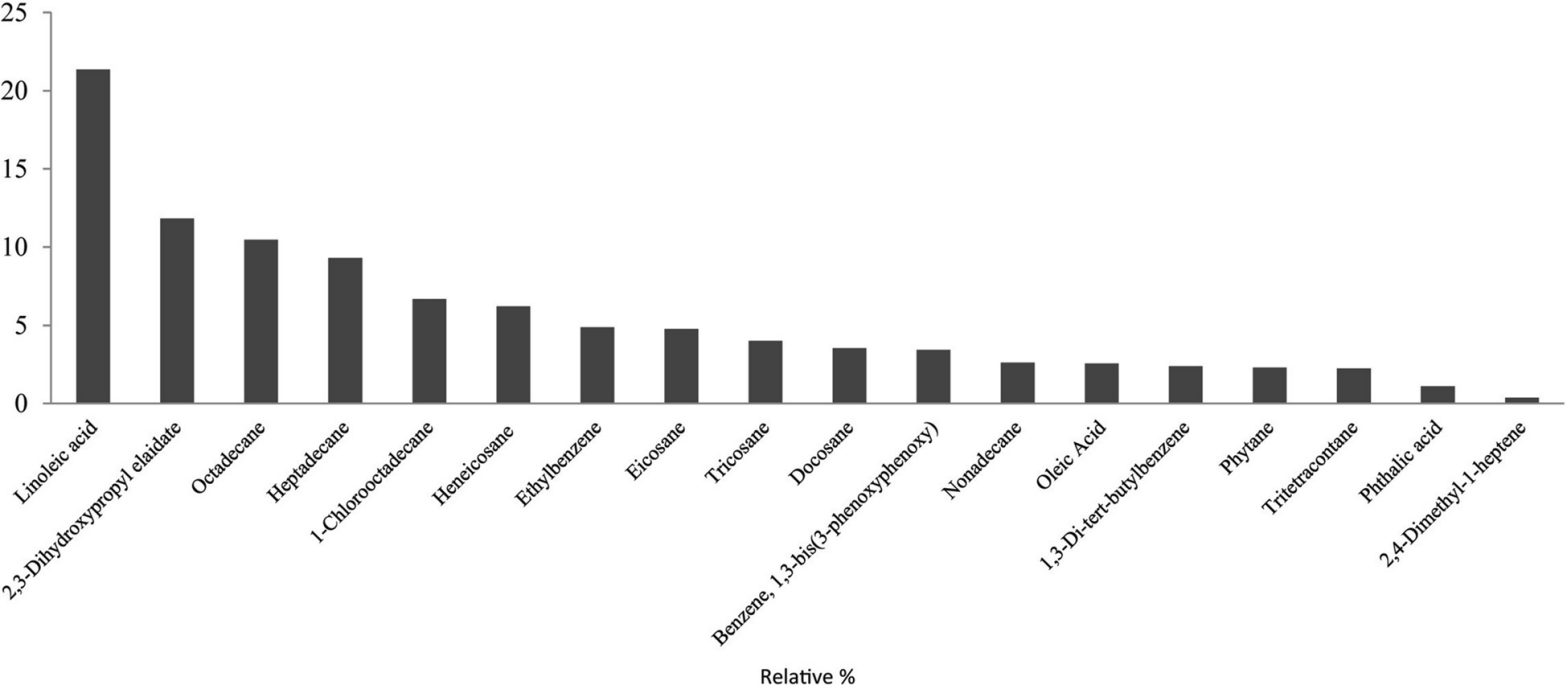
Volatile compounds detected in *L. rhinocerus* and their relative percentages

Five major groups, including alkanes (53 %; estimated weight fraction of 53 mg) and fatty acids (36 %), were detected (Fig. 1). Overall, eighteen constituents were extracted by the five solvents (petroleum ether, diethyl ether, hexane, ethyl acetate and methanol) (Fig. 2).

The highest numbers of constituents (eight compounds) were reported from non-polar (hexane) extraction followed by ethyl acetate (6 compounds), petroleum ether (5 compounds), diethyl ether (3 compounds) and methanol (2 compounds) extractions (Table 1). The constituent with the greatest proportion in *L. rhinocerus* hot water extract was linoleic acid (21.35 %), followed by 2,3-dihydroxypropyl elaidate (11.82 %) and octadecane (10.47 %). The chemical structures and molecular formula for the detected volatile constituents are described in Table 2.

**Table 1:** Volatile compositions of *L. rhinocerus* following extraction using various solvents and their respective retention times, RT (min)

Compounds	P.E	R.T	D.E	R.T	HX	R.T	E.A	R.T	MTL	R.T	Relative %
Octadecane	✓	10.31			✓	13.61	✓	9.43			10.47
Heptadecane	✓	12.76			✓	12.79	✓	8.99			9.29
Benzene, 1,3-bis (3-phenoxyphenoxy)	✓	3.18									3.43
Phytane	✓	9.86									2.30
Docosane	✓	12.05									3.54
Phthalic acid			✓	9.81							1.10
2,4-Dimethyl-1-heptene			✓	3.88							0.36
1,3-Di-tert-butylbenzene			✓	6.77	✓	6.76					2.38
Heneicosane					✓	14.5					6.22
Tricosane					✓	15.47					4.00
1-Chlorooctadecane					✓	16.48					6.69
Tritetracontane					✓	10.83					2.25
Eicosane					✓	10.84	✓	10.32			4.77
Nonadecane							✓	9.86			2.61
2,3-Dihydroxypropyl elaidate							✓	28.89			11.82
Oleic acid							✓	27.54			2.55
Linoleic acid									✓	3.20	21.35
Ethylbenzene									✓	4.08	4.87
Total percentage											100.00

*PE* petroleum ether, *DE* diethyl ether, *HX* hexane, *EA* ethyl acetate, *MTL* methanol

**Table 2:** Chemical structures of the major volatiles present in *L. rhinocerus*

Compound	Molecular formula	Chemical structure
Linoleic acid	C18H32O2	
2,3-Dihydroxypropyl elaidate	C21H40O4	
Octadecane	C18H34	
Heptadecane	C17H36	
1-Chlorooctadecane	C18H37Cl	
Heneicosane	C21H44	
Ethylbenzene	C8H10	
Eicosane	C20H42	
Tricosane	C23H48	
Docosane	C22H46	
Benzene, 1,3-bis(3-phenoxyphenoxy)	C30H24O4	
Nonadecane	C19H40	
Oleic Acid	C18H34O2	
1,3-Di-tert-butylbenzene	C14H22	
Phytane	C20H42	
Tritetracontane	C43H88	
Phthalic acid	C8H6O4	

### Inhibition effects of *L. rhinocerus* on airway inflammation

A preliminary study was conducted prior to the present investigation to optimize *L. rhinocerus* dosages and the dose used in the sensitization protocol that was suitable for the anti-asthmatic study. Based on this finding, a dose of 500 mg/kg of *L. rhinocerus* is more effective in reducing asthma-related parameters when compared to low (125 mg/kg) and medium (250 mg/kg) dosages (unpublished data) and was therefore selected for the study. A previous toxicity study indicated that 1000 mg/kg of *L. rhinocerus* extract fed orally to rats did not cause any adverse effects, and it is therefore considered to be safe [23]. The sensitization method was modified from a previous study [20] based on similar preliminary optimization study, hence i.p. sensitization with the additional adjuvant of Bordetella pertussis and 7 days of challenge or treatment was suitable to establish the expected allergic model.

Our study targeted serum IgE detection, while Th2 cytokine detection was conducted in BALF. As in asthmatic models, airway obstructions and difficulties in breathing are assumed to result from the responses of immunoglobulins and cytokine levels, which lead to worsening of asthma symptoms. Eosinophils are also found in large numbers within the submucosa and epithelium in the allergic model. Most allergic mediators are stimulated by IgE and are released into the blood stream in response to inflammatory reactions [9]. In addition, BALF biofluid is widely accepted as a reliable sample with which to determine the composition of secreted pulmonary proteins and the products of activated cells. Therefore, in this study we aimed to investigate OVA-induced IgE detection in serum, while Th2 cytokines were detected in BALF.

**Fig. 3:**
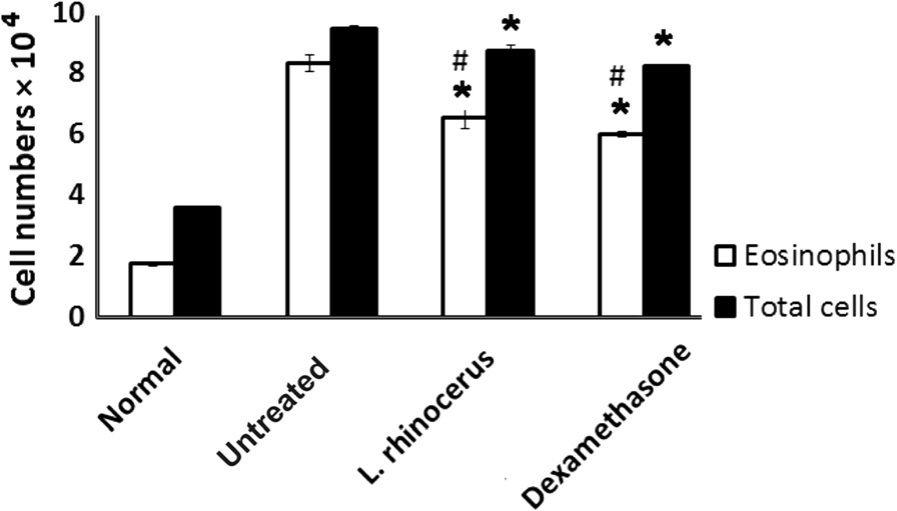
The effect of *L. rhinocerus* extract on the recruitment of inflammatory cells in BALF. Normal; untreated, OVA-sensitized/challenged rats; *L. rhinocerus* extract + OVA-sensitized/ challenged rats; dexamethasone + OVA-sensitized/ challenged rats. Values are expressed as the means ± SD (*n* = 5/group). *Significantly different from untreated. #Significantly different within treatment

High numbers of inflammatory cells in BALF indicate inflammation and an allergic condition. The level of inflammatory cell eosinophil infiltration in BALF was significantly reduced in the *L. rhinocerus* and dexamethasone groups compared to the untreated group (Fig. 3). However, the reduction observed with dexamethasone was slightly more prominent when compared to *L. rhinocerus.* The untreated group had the highest level of eosinophils, indicating marked induction of allergic asthma. The level of eosinophils in the untreated group was also significantly elevated when compared to the normal group.

**Fig. 4:**
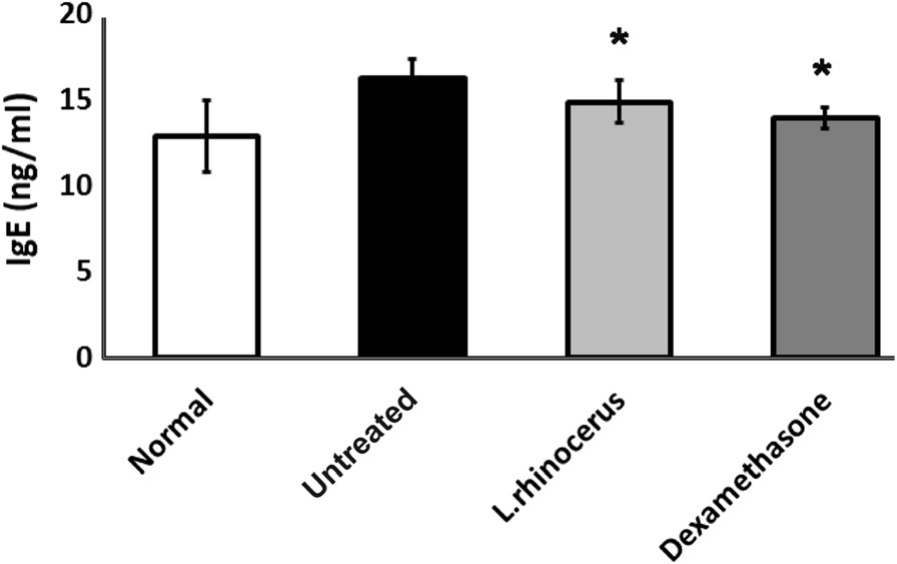
The effect of *L. rhinocerus* extract on serum IgE levels. Normal; untreated, OVA-sensitized/challenged rats; *L. rhinocerus* extract + OVA-sensitized/challenged rats; dexamethasone + OVA-sensitized/ challenged rats. Values are expressed as the means ± SD (*n* = 5/group). *Significantly different from untreated

Similarly, there was also a significant reduction in the mean serum IgE levels among animals receiving *L. rhinocerus* or dexamethasone when compared to the untreated group (Fig. 4). The reduction in the levels of IgE and Th2 cytokines indicate successful asthmatic treatment. In comparison, the IgE levels in the untreated group were significantly elevated when compared to the normal group.

**Fig. 5:**
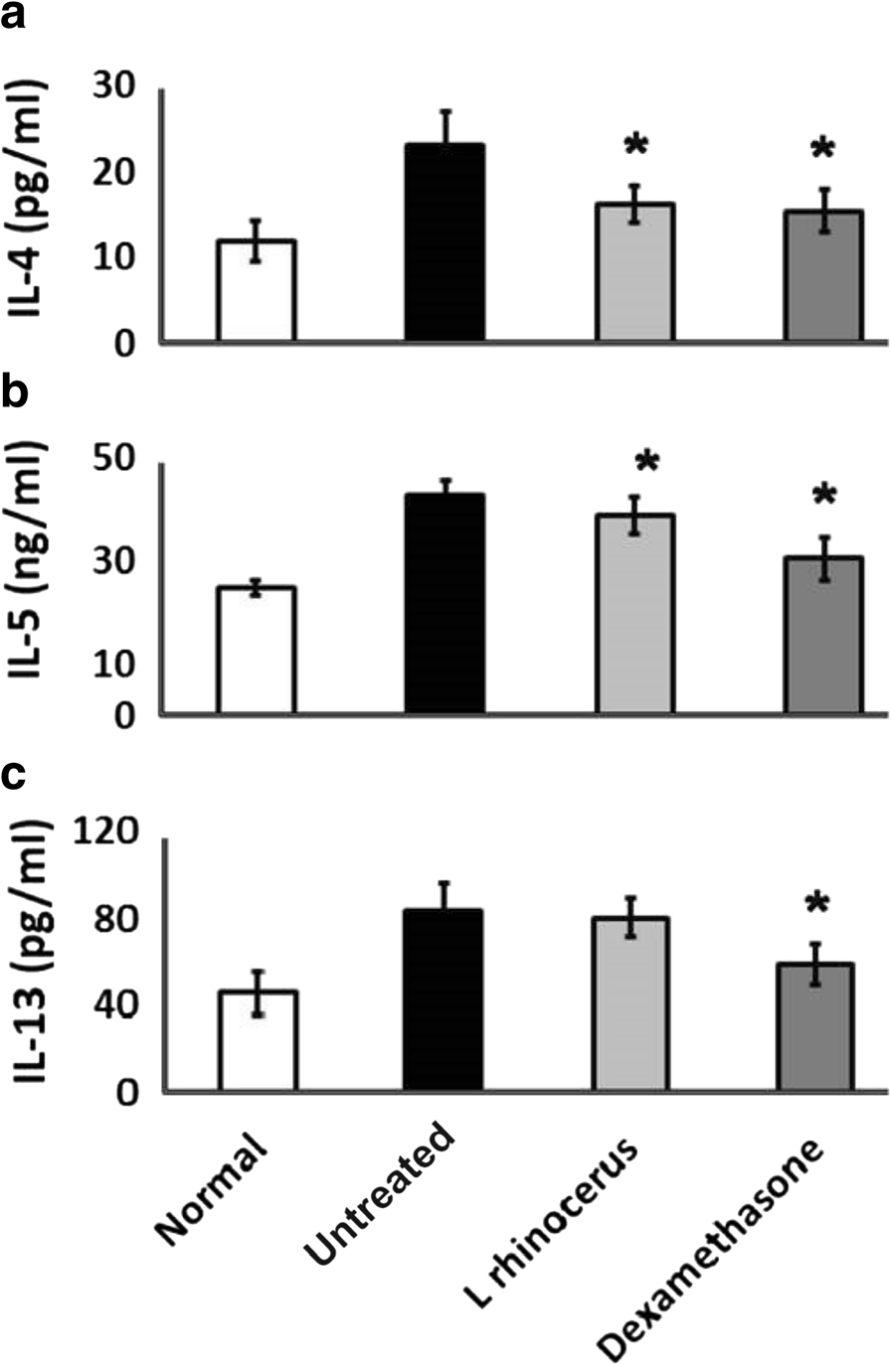
The effect of *L. rhinocerus* extract on the cytokines (**a**) IL-4, (**b**) IL-5 and (IL-13) levels in BALF. Normal; untreated, OVA-sensitized/challenged rats; *L. rhinocerus* extract + OVA-sensitized/challenged rats; dexamethasone + OVA-sensitized/ challenged rats. Values are expressed as the means ± SD (*n* = 5/group). *Significantly different from untreated

The levels of IL-4 (Fig. 5a) and IL-5 (Fig. 5b) were significantly reduced in the *L. rhinocerus* and dexamethasone groups when compared to the untreated group. There was also a reduction in the IL-13 level among animals receiving *L. rhinocerus* and dexamethasone. However, the reduction in IL-13 observed among animals receiving *L. rhinocerus* was not statistically significant when compared to the untreated group (Fig. 5c). The levels of all Th2 cytokines in the untreated groups were significantly elevated when compared to the normal group.

**Fig. 6:**
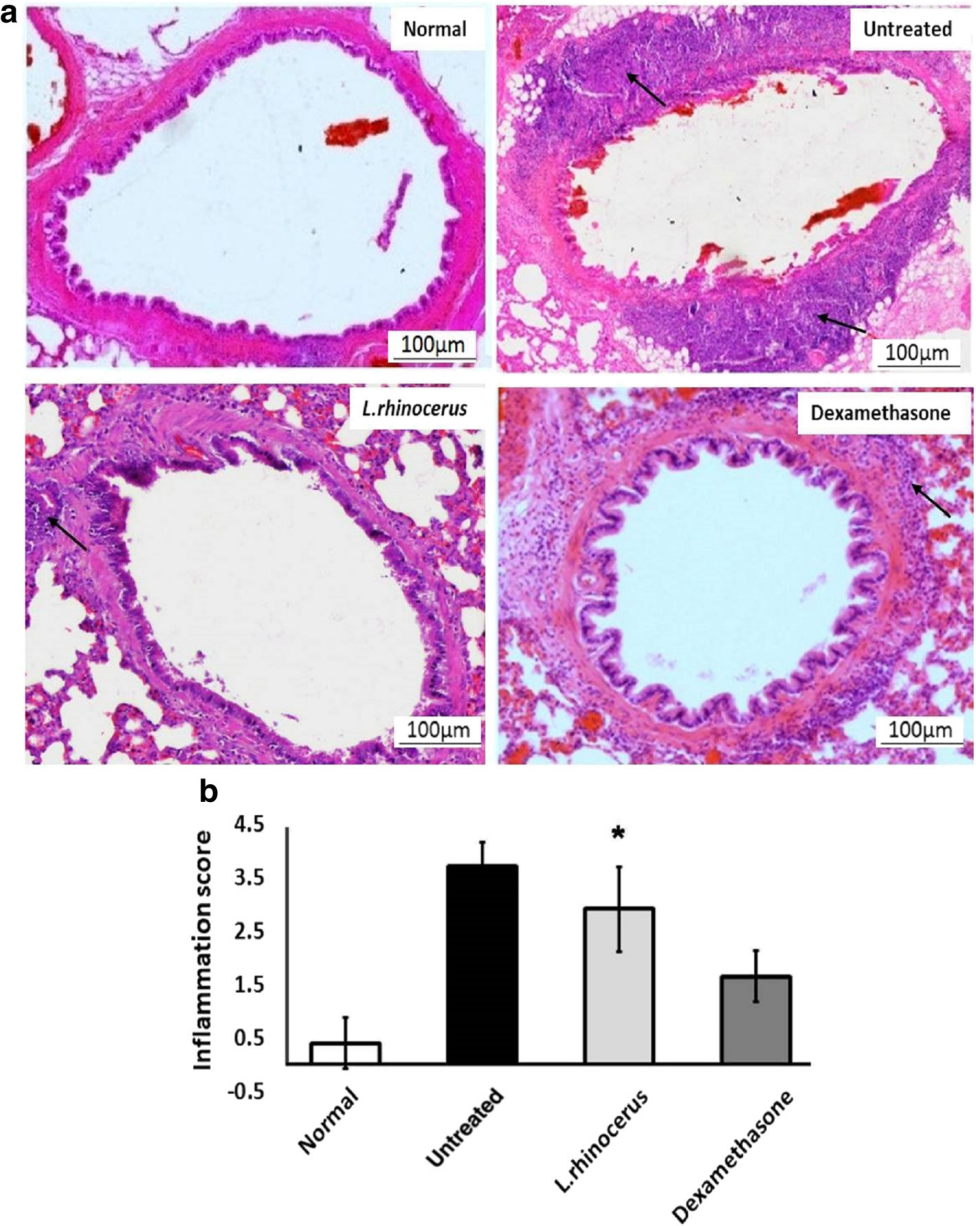
The effect of *L. rhinocerus* on airway inflammatory leukocyte infiltration in the peribronchiole region and perivascular connective tissue. (**a**) Magnification 50×. Black arrows indicate the presence of infiltration with eosinophils/leukocytes surrounding the bronchiole. (**b**) Quantitative analysis on the inflammation score with a subjective scale of 0–4 used to assess leukocyte infiltration in the lungs

Haematoxylin and eosin staining was performed on the lung tissues to analyze the effects of *L. rhinocerus* extract on the histological feature of asthma. The lung tissue section of ovalbumin-induced asthmatic rats was characterized by the presence of dense peribronchial inflammation due to leukocyte infiltration when compared with the normal tissue (Fig. 6a). Substances that can attenuate this inflammation indicate that they are effective anti-asthmatic agents. Histopathological findings further confirmed that there was significant reduction in the severity of eosinophil cell inflammation with significantly reduced cells forming eosinophil layers/rings in both the *L. rhinocerus* and dexamethasone groups. In addition, the inflammation score indicated that both the *L. rhinocerus* and dexamethasone groups showed significantly reduced inflammation when compared to the untreated group (Fig. 6b).

## Discussion

Our study is the first to elucidate the composition of hot water extract of *L. rhinocerus* widely used in the traditional treatment of asthma. Alkanes were the major group found to be present, while linoleic acid was the major constituent detected. To our knowledge, our study is also the first to confirm the airway inhibition effects of *L. rhinocerus.*

The GC-MS analysis was conducted following a sequential extraction method that employs both polar and non-polar properties of solvents in separating compounds based on polarity. In this study, a simple liquid-liquid extraction method is beneficial because it does not involve a heating process, which minimizes damage to the volatiles and can prevent the formation of other artefacts [24, 25]. This sequential extraction conserves the amount of volatiles as well as selectively improves the process and the recovery of different types of extracts from analogous materials. The compounds were selected based on spectral library matching to the NIST and Wiley libraries.

Most of the compounds detected were from the alkane group. In comparison, a study of other types of medicinal mushrooms such as *Termitomyces* sp. and *Termitomyces microcarpus* prepared from petroleum ether crude extracts reported the presence of small amounts of octadecane (0.64 %) and heptadecane (1.01 %) [26], which are alkanes. Another researcher reported the presence of octadecane (0.15 %) in *Trichodermaharzianum* following GC-MS analysis [27]. However, our study detected the presence of higher amounts of octadecane (10.47 %) and heptadecane (9.29 %) in *L. rhinocerus*. Similar to our study, a study on *Agaricus bisporus* comparing different strains of mushrooms (strains 310 and 342) reported high amounts of non-polar compounds such as octadecane (13.8 %) and nonadecane (14.4 %) in addition to eicosane and heptadecane [28]. In addition, several studies [28–30] have reported fatty acids to be the predominant constituent in mushrooms. Specifically, linoleic acid has been detected to be the major constituent (~65 %) of *Schizophyllum commune* and *Lentinus edode*s [31], which is also reported in our study as the most common compound (21.35 %). Similar to our study, the fatty acid profile of several *Tricholoma* species, such as *T. portentosum* and *T. terreum*, also revealed the presence of linoleic acid (∼28 %) [32].

Linoleic acid is a known precursor of 1-octen-3-ol (alcohol of fungi), which functions as the principal aromatic compound in most fungi [33–35]. A study on *L. rhinocerotis* comparing mycelium of shaken cultures and static cultures revealed the presence of 2.3-dihydroxypropyl elaidate compound (0.27–0.28 %) [36]. The presence of a similar compound was also detected in the spore lipids of *Ganoderma lucidum* (0.07 %) [37]. In our study, 2,3-dihydroxypropyl elaidate was reported to be present at approximately 11.82 % and constituted the second most common compound detected. In comparison, linoleic acid was compared in different parts of *A. bisporus* mushroom and was reported to be present in 12.2 % of the volume of the whole fresh sample, in 1.5 % of the stalks and in 0.8 % of the gills [38].

Linoleic acid was present in varying concentrations in different types of mushrooms, and unique properties were observed. In fact, such variations are key to understanding the aromatic and other medicinal properties. Nonetheless, GC-MS analysis of *L. rhinocerus* revealed compounds that were detectable and comparable with other mushrooms types and plants [29, 39, 40], making them both similar and different to plants of numerous species and genus.

This study is the first to report on the inhibition effects of *L. rhinocerus* on airway inflammation in asthmatic model. Asthmatic model of Sprague Dawley rat was used in the study and dexamethasone was used as a positive control as this compound is commonly used as a potent inhibitor of airway inflammation and remodelling. The administration of corticosteroids has been demonstrated to inhibit structural changes associated with airway fibrosis in animal models and is therefore widely utilized as a control in asthma studies [41]. In the context of the experimental design, all rats in this study (except for the normal group) underwent sensitization on days 1 (primary sensitization) and 14 (secondary sensitization). The purpose of an additional sensitization step on day 14 is to ensure that the levels of airway eosinophils do not revert back to the baseline, as well as to avoid the development of tolerance. In addition, these sensitizations were coupled with OVA along with alum and *Bordetella pertussis* to enhance Th2-dominated responses [42].

Generally, the activation, growth and differentiation of eosinophils are conducted by Th2-dominated responses [43]. It is reported that cytokines secreted by Th2 cells can help in the recruitment and activation of eosinophils in the nasal region. Eosinophil counts performed on BALF in this study revealed that treatment with hot water extract of *L. rhinocerus* has reduced the number of eosinophils to 6.54 × 10^4^ cells in BALF when compared to the untreated group, which contained 8.34 × 10^4^ cells; this could indicate attenuated severity and possible withdrawal in eosinophil infiltration along the submucosa and epithelium regions. Groups of rats receiving hot water extract of *L. rhinocerus* and dexamethasone treatment had also significantly decreased the numbers of total inflammatory cells in BALF. These decreases are correlated with the level of cellular infiltration. Furthermore, the deterioration of inflammatory cell numbers in BALF was confirmed by lung tissue histology, thus possibly be valuable in controlling the inflammatory processes underlying exacerbation of allergic asthma. This result is in accordance with the previous study that demonstrated the reduction of eosinophils in OVA-treated animals after treatments with herbal extract [8].

An early finding indicated that in sensitized individuals, the nasal mucosa contains high level of IgE-binding mast cells [44]. Upon sensitization, increasing numbers of IgE-coated mast cells traverse the epithelium. During the phase of allergen exposure or challenge, the IgE-coated mast cells recognize the allergens deposited at mucosal regions, and the mast cells then degranulate. This leads to the release of mediators such as histamines and leukotrienes, which are responsible for blood vessel leakage to stimulate the sensory nerves leading to congestion, airway inflammation and obstruction as well as systemic reflexes such as sneezing [45]. Thus, reducing IgE levels has been the major focus in combating asthma. Accordingly, animals receiving hot water extract of *L. rhinocerus* had significantly reduced IgE levels (14.88 ng/ml) when compared to the untreated group (16.29 ng/ml). A recent study also reported that another type of mushroom polysaccharide from *A. camphorata* has similar anti-asthmatic effects shown by reducing IgE levels in an animal model of asthma [46].

In our study, it was noted that hot water extract of *L. rhinocerus* significantly reduced IL-4 levels in BALF. This is supported by a study using murine allergen challenged models where intranasal chitin polysaccharide components of fungal cell decreased IL-4 (16.22 pg/ml) production compared to untreated group (23.07 pg/ml) *in vivo* [47, 48]. Moreover, Th2 cytokine concentration levels of IL-5 (38.82 ng/ml) and IL-13 (80.54 pg/ml) were also reduced when hot water extract *L. rhinocerus* was used to treat sensitized rats in comparison with untreated groups (42.79 ng/ml and 83.69 pg/ml), respectively. Similarly, both IgE levels in the serum and Th2 cytokines in BALF were also reduced by dexamethasone. However, the reduction observed in the IL-13 levels due to *L. rhinocerus* administration was not significant, which may illustrate the varying effects of *L. rhinocerus* extract on IL-13 regulation. Interestingly, IL-13 has been reported to potentially have independent roles from eosinophils, IgE, IL-4 and IL-5 [49]. It has been reported that the production and high activity of eosinophils, the hallmark of asthma, was not affected despite inhibition of IL-13 [49]. However, the potential independent role of IL-13 in prompting the complete allergic asthmatic phenotype has been underlined. In a study on IL-13-dependent airway hyper responsiveness (AHR), it was revealed that the mechanisms are independent of IgE and eosinophils in mice [6].

The lung histopathology study further confirmed how structural changes or responses involving lung tissues further contribute to asthma. Indications of such as high leukocytes or eosinophil infiltration at regions around the peribronchial and perivascular space in the bronchioles are important keys that point to such a severe condition [22]. Consistent with previous findings regarding immunoglobulin and Th2 cytokines, both the hot water extract of *L. rhinocerus* and dexamethasone were noted to reduce the number of eosinophil cells infiltrating into lungs tissue. A similar histological study on *A. Camphorata* also reported attenuation of lung inflammatory leukocytes in OVA-sensitized animals [45]. Relief of bronchoalveolar inflammation in a murine model of airway sensitivity has also been reported in a histology study that used another type of mushroom i.e., *Ganoderma tsugae* (*G. tsugae)* as a possible treatment for allergic asthma [50].

Several findings further confirm that immune disorders are attributable to the collapse of the system that controls the balance between levels of Th1 to Th2 cells. In fact, diseases such as leprosy, allergic asthma and multiple sclerosis are associated with aberrant Th1 and Th2 polarizations. Th2 cells are also strongly implicated in atopy and allergic inflammation. *A. camphorata* polysaccharides, for instance, are potential inducers of Th1-type cytokines such as tumour necrosis factor and interferon but not of Th2 cytokines, which have successfully been proven to reduce asthmatic conditions [46]. The importance in discovering and identifying new drugs that are safe and effective and have minimal side effects has made the medicinal properties of plants more valued and thereby more widely explored. Our study demonstrated that treatment of OVA-induced airway inflammation among Sprague Dawley rats treated with hot water extract of *L. rhinocerus* significantly reduced allergic asthma parameters i.e., IgE level, Th2 cytokines, eosinophil count and eosinophil infiltration in the lungs.

Various speculations denote the potential anti-asthmatic effects of *L. rhinocerus*. Investigating the roles of fatty acids in reducing asthma symptoms has been gaining high attention in studies of animal models as well as children [51, 52]. Interestingly, a previous chemical study on *L. rhinocerotis* revealed that major compounds such as fatty acids are present [36].

Polyunsaturated fatty acids (PUFA) have been found to have potential anti-inflammatory properties with fewer side effects, and they are claimed to be mildly beneficial for the treatment of allergic conditions such as asthma and atopic dermatitis [53]. In fact, previous studies [53, 54] have proven that PUFAs are closely associated with enhanced lung function and reduced asthma symptoms. It has been shown that n-3 PUFAs suppress a major mediator of asthma known as leukotriene synthesis [51]. The role of such fatty acids is also associated with the modulation of prostaglandin metabolism, which is the primary cause of exacerbated conditions of allergic asthma. It has been proven that the anti–asthmatic potential of *Zanthoxylum bungeanum* is due to the presence of some phytochemical compounds, specifically those rich in linoleic acids [54]. Therefore, it is plausible that the presence of high concentrations of linoleic acid in *L. rhinocerus* which is also one of the main key constituents, contributes to the anti-asthmatic effect. Nevertheless, this should be confirmed in a further study.

It should be noted that there may be differences in the type of detectable volatile compounds present in fresh *L. rhinocerus* in contrast to the cultivated sample used. In addition, the spectral library matching performed in this study during the detection of volatile compounds provides preliminary presumptive data; therefore, future studies that contribute to the utilization of authentic standards should be conducted to further confirm our findings*.* Confirming the presence of compounds with long-chain fatty acids can be further supported by additional methylation steps as is also recommended by Mendez et al. [55]. Moreover, this study only focused on volatile compounds of *L. rhinocerus* as these compounds are reported to be the intermediate or end products of diverse metabolic pathways of various classes [27]. Identification of non-volatile and semi-volatile organics, which are more suitable for investigation using liquid chromatography/mass spectrometry, was not performed at this point of the study.

## Conclusion

Alkanes were the major group present in hot water extract of *L. rhinocerus*, while linoleic acid was the major constituent detected. *L. rhinocerus* showed anti-asthmatic effects *in vivo* as confirmed by the decreased levels of IgE and Th2 cytokines responsible for eosinophils recruitment, as well as the attenuation of eosinophil infiltration in the lungs. Thus, *L. rhinocerus* has the potential to be a promising alternative or adjuvant to the current drugs used for the management of allergic asthma.

## Abbreviations

BALF, bronchoalveolar lavage fluid; GC-MS, Gas chromatography mass spectrum; Ig, immunoglobulin; IL, interleukin; OVA, ovalbumin; TIC, total ion current.
